# Congenital Absence of Left Circumflex Artery Detected by Computed Tomography Coronary Angiography: A Case Report

**DOI:** 10.1155/2012/204657

**Published:** 2012-07-09

**Authors:** Keerati Hongsakul, Ruedeekorn Suwannanon

**Affiliations:** Department of Radiology, Faculty of Medicine, Prince of Songkla University, 15 Kanchanavanit Road, Hat Yai 90110, Thailand

## Abstract

The congenital absence of the left circumflex artery (LCx) is a very rare congenital anomaly of coronary arteries, but it is benign. Currently, the best modality for the diagnosis of coronary anomalies is computed tomography coronary angiography (CTCA). We report a case of congenitally absent LCx with an atypical chest pain.

## 1. Introduction

Congenital anomalies of coronary arteries are a group of disease, which is infrequently found. Their prevalence has been reported to range from 0.6% to 1.3% [1]. Most clinical manifestations are benign and asymptomatic. However, about 20% of cases manifest significant clinical symptoms [1]. The congenitally absent left circumflex artery (LCx) is a very rare anomaly, and not many cases have been reported. In the past, this condition was mostly detected incidentally by coronary angiography. Recently, more advanced technology, for example, the use of computed tomography coronary angiography (CTCA), has proven better in the evaluation of coronary anomalies because it is faster, safer, and highly accurate [2]. We report a case of absent LCx with superdominant right coronary artery (RCA) detected by the CTCA.

## 2. Case Presentation

 A 52-year-old Thai man presented with acute chest tightness and palpitation while rubber farming for 1 week. He had a history of hypertension and heavy smoking for 10 years. He was admitted at a local hospital, and the results of the complete blood count and lipid profile showed within normal limits. His electrocardiogram showed a normal sinus rhythm with Wolff-Parkinson-White syndrome, and his clinical signs did not improve, so he was referred to our hospital. His clinical condition was suspected to be due to coronary arterial disease by a cardiologist. However, his exercise stress test was inconclusive, so he was further investigated by CTCA.

**Figure 1:**
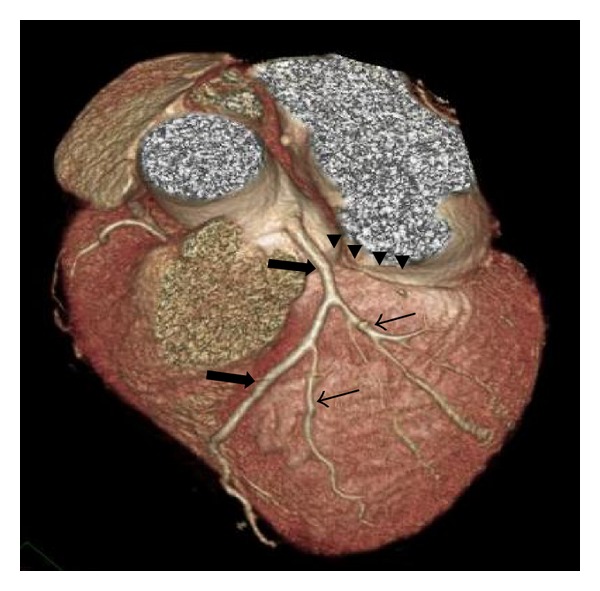
Top view three-dimensional computed tomography scan shows absent left circumflex artery in the atrioventricular groove (arrowed heads) with single left anterior descending artery (thick arrows) and prominent diagonal branches (thin arrows).

**Figure 2:**
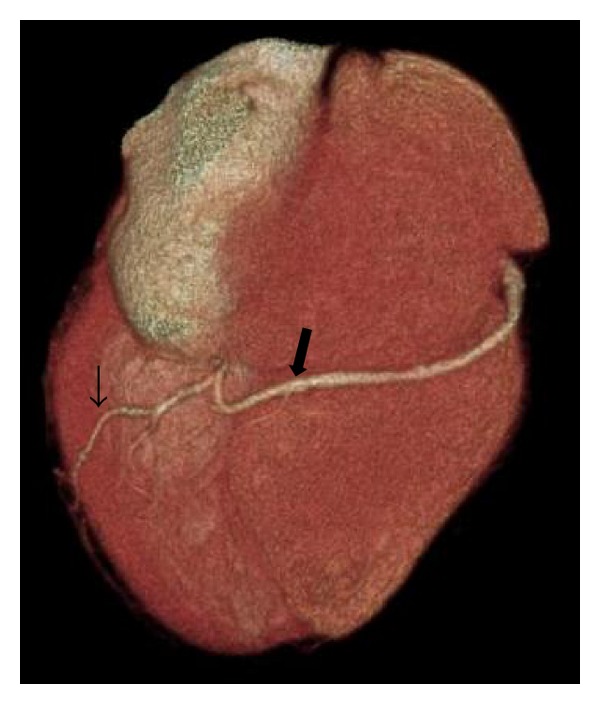
Inferior view of three-dimensional computed tomography scan shows superdominant right coronary artery (large arrow) to supply posterior wall (small arrow).

**Figure 3:**
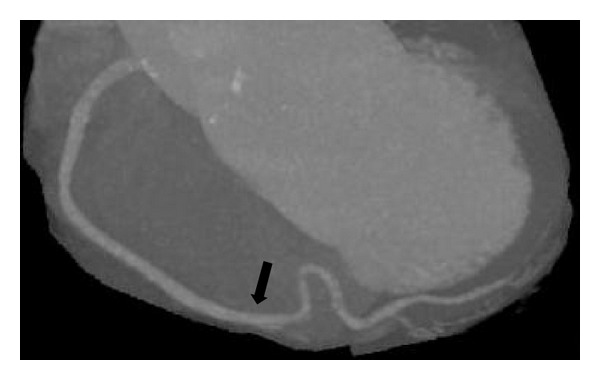
A maximal intensity projection image shows overall course of superdominant right coronary artery (arrow).

 The 64-slice CTCA findings showed no calcified coronary artery walls, and the calcium score was zero. A completely absent LCx was observed, which was associated with multiple enlarged diagonal branches of the left anterior descending artery (LAD) to supply the lateral wall of the left ventricle (Figure 1). Also seen was the superdominant RCA with its posterolateral branch continuing into the territory of the LCx (Figures 2 and 3). No significant stenosis was detected. The patient was treated with antiarrhythmic drug (amlodipine 2.5 mg/day) and scheduled for follow-up visits at his district hospital.

## 3. Discussion

 In the past, congenital anomalies of coronary arteries were incidentally diagnosed by conventional coronary angiogram; however, this is an invasive technique. Selective angiogram may find it difficult to determine the origin of an artery in the case of anomalous arterial origin. Moreover, its interpretation may also be difficult in cases of anomalous course and origin. For example, in our case, it may be difficult to differentiate between the congenital absence and total occlusion diagnoses based on the angiogram findings. Today, a more advanced technology, CTCA, is better for detecting congenital anomalies, because it is widely available, noninvasive and is characterized by a rapid acquisition of results [2]. In addition, this technique is highly accurate for the evaluation of congenital coronary artery anomalies because it can present a three-dimensional image and demonstrate the anatomical origin, course, and termination of coronary artery anomalies as well as define their relationship to other cardiac and noncardiac structures. Furthermore, these overviews can help the surgeon to evaluate the operation planning before bypass surgery. Shi et al. [3] have reported on the accuracy of multidetector computed tomography (MDCT) to evaluate coronary artery anomalies. In this study, MDCT and coronary angiography were evaluated in a blinded fashion for their accuracy of anomalous artery origin and path detection, and the results were compared in a secondary consensus. One hundred percent of coronary anomalies were correctly displayed on MDCT. Coronary angiography alone achieved a correct identification of the abnormality in only 53% of cases (*P* = 0.016). Kacmaz et al. [4] have reported on the sensitivity of MDCT in patients who had a coronary artery anomaly demonstrated by conventional coronary angiography. The result was a 100% diagnostic sensitivity for the detection of the origin and course of the anomalous coronary artery. We agree with Kacmaz et al. [4] and Baskurt et al. [5] that CTCA should be a first-choice imaging modality for the investigation of known or suspected coronary artery anomalies. Although, CTCA is beneficial for detecting coronary artery anomalies, it also suffers from disadvantages linked to patient exposure to ionizing radiation and reception of contrast agents that may potentially induce nephrotoxicity and allergy.

The congenitally absent LCx is an extremely rare anomaly of coronary arteries; a small number of cases have been reported. Its incidence was reported by Yamanaka and Hobbs to have a frequency of only 0.003% in patients undergoing coronary angiography [1]. Another report by Srinivasan et al. [2] indicated this condition in only 0.067% of 1495 patients undergoing MDCT. In our opinion, the incidence of this condition may be increasing due to the availability of a noninvasive tool, such as, CTCA that has experienced a wider use in helping to evaluate coronary arterial disease.

 The congenital absence of the LCx results from the failure of LCx development in the left atrioventricular groove. However, some authors believe that this condition is not a real congenital absent anomaly so it is defined as anomalous origin of the LCx from distal RCA [6, 7]. Almost always this anomaly is defined as a benign condition and has no significant clinical symptom. However, it can cause angina-like symptoms, particularly on exertion. Most of reported cases of this condition are claimed to have experienced chest pain on exertion; our case did too. The etiology of this angina-like symptom is not exactly known. One hypothesis that is accepted as an explanation of the pathophysiology of congenitally absent LCx is steal phenomenon [8–10]. This phenomenon causes an increased arterial supply to the LCx territory and results in transient ischemia of other coronary arterial territories. The detection of congenitally absent LCx is necessary because some cases have clinical symptoms mimicking coronary syndrome.

 The imaging findings of congenitally absent LCx show no demonstrable LCx in the left atrioventricular groove. This finding is associated with the superdominant RCA continuing into the LCx territory and prominent multiple diagonal branches from the LAD to supply the lateral wall of the left ventricle, as seen in many literatures [2–8] and also in our report.

 In conclusion, the congenital absence of the LCx is an extremely rare anomaly of coronary arteries. CTCA is the best modality to detect congenital coronary anomalies because it is fast, safe, and highly accurate.
